# Minimally Invasive Spleen‐Preserving Distal Pancreatectomy in Obese Patients: Factors Related to Clinically Relevant Pancreatic Fistula

**DOI:** 10.1002/rcs.70091

**Published:** 2025-07-08

**Authors:** Fabio Ausania, Filippo Landi, Carolina González‐Abós, John B. Martinie, Dionisios Vrochides, Matthew Walsh, Shanaz M. Hossain, Steven White, Viswakumar Prabakaran, Laleh G. Melstrom, Yuman Fong, Giovanni Butturini, Laura Bignotto, Valentina Valle, Yuntao Bing, Dianrong Xiu, Gregorio Di Franco, Francisco Sanchez‐Bueno, Nicola de'Angelis, Alexis Laurent, Giuseppe Giuliani, Graziano Pernazza, Alessandro Esposito, Roberto Salvia, Francesca Bazzocchi, Ludovica Esposito, Andrea Pietrabissa, Luigi Pugliese, José Rios, Andrea Coratti, Luca Morelli, Pier C. Giulianotti

**Affiliations:** ^1^ Department of HBP Surgery and Transplantation Hospital Clinic Barcelona Institut d’Investigacions Biomèdiques August Pi i Sunyer (IDIBAPS) University of Barcelona (UB) Barcelona Spain; ^2^ Division of HPB Surgery Department of Surgery Carolinas Medical Center Charlotte North Carolina USA; ^3^ HPB Surgery Department Digestive Disease and Surgery Institute Cleveland Clinic Cleveland Ohio USA; ^4^ Freeman Hospital NHS Newcastle‐Upon‐Tyne UK; ^5^ Division of Surgical Oncology Gastrointestinal Disease Team City of Hope Medical Center Duarte California USA; ^6^ Department of HBP Surgery P. Pederzoli Hospital Peschiera del Garda Italy; ^7^ Division of General Minimally Invasive and Robotic Surgery Department of Surgery University of Illinois at Chicago Chicago Illinois USA; ^8^ Department of General Surgery Beijing Third Hospital Beijing China; ^9^ Division of Translational and New Technologies in Medicine and Surgery General Surgery Department University of Pisa Pisa Italy; ^10^ Department of HBP Surgery Virgen de la Arrixaca Hospital Murcia Spain; ^11^ Department of Digestive HBP Surgery and Liver Transplantation Henri Mondor Hospital APHP Creteil France; ^12^ Division of General and Minimally Invasive Surgery Misericordia Hospital Grosseto Italy; ^13^ General and Robotic Surgery Department San Giovanni Hospital Rome Italy; ^14^ HBP Surgery Department Policlinico G.B. Rossi Hospital Verona Italy; ^15^ Department of HBP Surgery IRCCS Casa Sollievo Della Sofferenza Hospital Foggia Italy; ^16^ Department of HBP Surgery Policlinico S. Matteo Hospital Pavia Italy; ^17^ Department of Clinical Pharmacology Hospital Clinic and Medical Statistics Core Facility Institut d’Investigacions Biomèdiques August Pi i Sunyer (IDIBAPS) Biostatistics Unit Faculty of Medicine Universitat Autònoma de Barcelona (UAB) Barcelona Spain

**Keywords:** laparoscopic distal pancreatectomy, obesity, postoperative pancreatic fistula, robotic distal pancreatectomy, spleen‐preservation pancreatectomy

## Abstract

**Background:**

Minimally invasive spleen‐preserving distal pancreatectomy (MI‐SPDP) provides superior outcomes compared with open surgery, with robotic techniques showing better short‐term results than laparoscopic techniques, particularly in obese patients. This study aimed to evaluate the impact of the surgical approach on postoperative pancreatic fistula (POPF) incidence in obese patients undergoing MI‐SPDP.

**Methods:**

A retrospective analysis of obese patients from 16 international centres compared robotic (R‐SPDP) and laparoscopic (L‐SPDP) approaches. Perioperative outcomes and factors associated with clinically relevant POPF were analysed using univariate and multivariate methods.

**Results:**

Among 130 patients (57L‐SPDP, 73R‐SPDP), POPF incidence was significantly lower in the robotic group (15.1% vs. 42.1%; *p* = 0.001). The Comprehensive Complications Index was also lower (8% vs. 15%; *p* = 0.002). Laparoscopic approach (OR = 4.0), pancreatic body transection (OR = 2.6), and non‐stapler stump closure (OR = 3.2) were independently associated with higher POPF rates.

**Discussion:**

Robotic MI‐SPDP reduces POPF in obese patients. Transection at the pancreatic neck and stapler‐based closure can improve outcomes.

## 1 Introduction

Background: Spleen‐preserving distal pancreatectomy (SPDP) is performed to excise benign, premalignant, and low‐grade malignant lesions in the body and tail of the pancreas. The decision to preserve the spleen during left pancreatectomy involves balancing the risks of inadequate oncological clearance against the benefits of avoiding potential complications related to splenectomy, such as thrombocytosis, thromboembolism, and infections.

Minimally invasive spleen‐preserving distal pancreatectomy (MI‐SPDP) techniques, including robotic (R‐SPDP) and laparoscopic (L‐SPDP) approaches, are recognized as safe options for selected patients undergoing left pancreatectomy [1, 2, 3, 4]. The robotic approach has become increasingly popular for minimally invasive distal pancreatectomy (MIDP), with previous studies indicating that it is associated with a lower rate of conversion to open surgery and better postoperative outcomes compared to laparoscopy [5, 6].

A recent meta‐analysis encompassing 11 studies with 685 patients undergoing MI‐SPDP highlighted the advantages of R‐SPDP over L‐SPDP, including a lower risk of failure to preserve the spleen, reduced conversion to open surgery, decreased blood loss, and shorter hospital stays [7]. However, this study did not reveal significant differences in major postoperative complications or clinically relevant postoperative pancreatic fistula (POPF). Additionally, another systematic review of 20 studies comparing different SPDP techniques (vessel‐preserving vs. Warshaw technique) and including laparoscopic, robotic, and open approaches found no significant differences in major morbidity, including clinically relevant POPF [8, 9, 10].

We have previously demonstrated the superiority of the robotic approach over the laparoscopic approach in terms of lower conversion rates, fewer complications, and higher spleen preservation rates in obese patients undergoing MIDP [11]. Despite the known impact of a high body mass index (BMI) on postoperative morbidity and the increased incidence of POPF associated with MIDP [12, 13], there are no studies specifically comparing outcomes of R‐SPDP and L‐SPDP in obese patients.

In this study, we hypothesize that the robotic approach may reduce the incidence of clinically relevant POPF, and major postoperative complications compared with laparoscopy in obese patients undergoing MI‐SPDP. The primary aim of this study was to compare the rates of POPF and other short‐term outcomes between patients with a BMI ≥ 30 undergoing R‐SPDP versus L‐SPDP, and to identify factors associated with clinically relevant POPF.

## 2 Material and Method

### 2.1 Study Design

Approval of the institutional review board of our Institution (CEIm, Comité de Ética de la Investigación con medicamentos) with identification number HCB/2022/0438 was obtained. Based on our previous international cross‐sectional studies [11, 14], we performed a nested and dedicated study of patients with a planned spleen‐preservation in MIDP focussing on factors predictive of clinically relevant POPF (grade B/C of the ISGPS classification) [15].

This study is based on the retrospective analysis of a single database including all consecutive patients with obesity who underwent MI‐SPDP between January 2012 and January 2022 in 16 international centres. A specific pseudo‐anonymized database was generated by the coordinating centre (Hospital Clinic, Barcelona, Spain) from different data sources including the variables of interest collected by each centre. The study was approved by the institutional review board of any participating center and conducted in accordance with the ethical principles of the Declaration of Helsinki.

Inclusion criteria were as follows: (a) MIDP performed for any indication with planned spleen‐preservation (b), BMI ≥ 30 and (c) age > 18 years. Exclusion criteria were: (a) MIDP in nonobese patients (BMI < 30) without preoperatively planned spleen‐preservation and (b) open surgery.

### 2.2 Data Collection

Demographic and clinical variables were analysed. Patient demographics including age, sex, BMI, American society of Anesthesiology (ASA) classification and comorbidities were recorded. Surgical characteristics, including indication of resection, anatomical data, type of surgery, indications for preoperative planning of MI‐SPDP, devices and other surgical technique details were also collected.

### 2.3 Preoperative Evaluation and Outcomes

All patients underwent pre‐operative abdominal computed tomography. A MI‐SPDP was planned by each center program in case of benign disease or low‐risk (pre)malignant disease classified as follows: well‐differentiated pancreatic neuroendocrine tumor (PNET), intraductal papillary mucinous neoplasm (IPMN), or other lesions with any grade of dysplasia. When a MI‐SPDP was planned, the reasons for unplanned splenectomy were registered. Conversion was defined as the need for a laparotomy to complete the operation in an instance of technical complexity or complications. A MI‐SPDP was considered successful only in case of technical achievement of spleen preservation regardless of the approach.

Methods of pancreatic stump closure were also recorded, includingthe use of an endostapler, suturing of the pancreatic stump, use of an ultrasonic device for pancreatic transection, and other methods. The type of stapler cartridge was not recorded. The use of sealant patches was also not included as there is no evidence of the impact of these materials on the development of POPF.

The main outcomes included any morbidity at 90 days, time to regular diet, hospital stay, readmission rate, operative time, conversion rate and blood loss. Postoperative complications were classified according to the ClavienDindo (CD) classification [16], and severe morbidity was defined as CD grade ≥ 3. The Comprehensive Complications Index (CCI) was used to assess morbidity in patients with multiple complications [17]. Data on the POPF rate and its grading were also collected [15].

### 2.4 Statistical Analysis

Quantitative variables are described as means ± standard deviations, and qualitative variables are described as absolute frequencies and percentages. For two‐sided group comparisons, the *χ* ^2^ test or Fisher's exact test were used for categorical variables, and the Mann‐Whitney *U* test was applied for continuous variables. Any variable identified as significant or with a value of *p* < 0.050 in the univariate analysis will be considered a candidate for the Cox regression multivariate analysis and the results will be presented as Odds Ratio with 95% confidence intervals. A value of *p* < 0.05 was considered statistically significant. SPSS version 26.0 (IBM, Armonk, NY, United States) and SAS version 9.4 (SAS Institute, Cary, NC, United States) were used for the statistical analysis.

## 3 Results

### 3.1 Demographics and Operative Characteristics

Out of 446 MIDP patients, 130 patients underwent a MI‐SPDP. Preservation of the spleen initially planned was achieved in 86 patients (66.2%): 39 out of 57 (68.4%) and in 47 out of 73 (64.4%) in the L‐SPDP versus R‐SPDP groups, respectively [11].

Table 1 shows the demographic and preoperative characteristics of both groups. The groups did not differ in terms of age, BMI, previous abdominal surgery, ASA status, cardiorespiratory comorbidity, smoking habit, or indication for surgery. Male sex was significantly more frequent in the L‐SPDP versus. R‐SPDP group (56% vs. 44%, *p* = 0.008), while preoperative diabetes was more frequent in the R‐SPDP than L‐SPDP group (22.2% vs. 7%; *p* = 0.026). There was no difference between groups in terms of tumour diameter and anatomical region of pancreas transection (Table 1).

**TABLE 1:** Robotic versus laparoscopic spleen‐preservation distal pancreatectomy in obese patients: demographics and pre‐operative characteristics.

Variables	Laparoscopic spleen‐preservation group (L‐SPDP)	Robotic spleen‐preservation group (R‐SPDP)	Overall	*p* value
	(*n* = 57)	(*n* = 73)	(*n* = 130)	
Age (yrs), mean (SD)	52 (15.4)	54.4 (14.2)	53.4 (14.8)	0.353
Male sex, *n* (%)	36 (56.3)	28 (43.8)	64 (49.2)	**0.008**
BMI (kg/m^2^), mean (SD)	34 (4.9)	34.4 (5.1)	34.3 (5)	0.664
ASA status, *n* (%)				
‐I	4 (7)	—	4 (3.1)	0.289
‐II	36 (63.2)	37 (50.7)	73 (56.1)	
‐III	17 (29.8)	35 (47.9)	52 (40)	
‐IV	—	1 (1.4)	1 (0.8)	
Previous abdominal surgery, *n* (%)	26 (45.6)	41 (56.2)	67 (51.5)	0.289
Cardiovascular disease, *n* (%)	17 (29.8)	25 (34.2)	42 (32.3)	0.567
Respiratory disease, *n* (%)	10 (17.5)	14 (19.2)	24 (18.5)	0.650
Diabetes, *n* (%)	4 (7)	16 (22.2)	20 (15.5)	**0.026**
Smoking, *n* (%)	5 (8.8)	14 (19.2)	19 (14.6)	0.133
Comorbidity > 1, *n* (%)	16 (28.1)	28 (38.4)	44 (33.8)	0.264
Indication of spleen preservation, *n* (%)				
‐IPMN low risk	2 (3.5)	8 (11)	10 (7.7)	0.260
‐Serous/mucinous cystadenoma	17 (29.8)	27 (37)	44 (33.8)	
‐PNET diameter < 2 cm	20 (35.1)	19 (26)	39 (30)	
‐Other	18 (31.6)	19 (26)	37 (28.5)	
Lesion location imaging, *n* (%)				
‐Neck	3 (5.3)	5 (6.8)	8 (6.2)	
‐Body	10 (17.5)	19 (26)	29 (22.3)	
‐Tail	41 (71.9)	41 (57.5)	82 (63.0)	
‐Diffuse (≥ 2 sites)	3 (5.3)	7 (9.6)	10 (7.7)	0.395
Tumour size imaging (mm), mean (SD)	36 (35)	29 (20)	32 (28)	0.191

*Note:* In bold: statistically significant difference.

Abbreviations: ASA, American society of anesthesiologists; BMI, body mass index; IPMN, intraductal papillary mucinous neoplasm; PNET, pancreatic neuroendocrine tumor; SD, standard deviation.

Operative characteristics and postoperative outcomes are shown in Table 2. In the R‐SPDP group 8.5% of patients had a Warshaw's technique (splenic vessel resection) versus 35.9% in the L‐SPDP group (*p* = 0.030). The preferred type of operation in each group was the Kimura technique (spleen vessels preservation) that was performed in 64.1% and 59.6% of patients in the L‐SPDP and R‐SPDP groups, respectively. Pancreatic transection was significantly more frequently performed at the isthmus within the R‐SPDP versus. L‐SPDP group (49.3% vs. 31.6%; *p* = 0.050) without differences regarding the method selected to close the pancreatic stump (Table 2).

**TABLE 2:** Robotic versus laparoscopic spleen‐preservation distal pancreatectomy in obese patients: operative characteristics and postoperative outcomes.

Variables	Laparoscopic spleen‐preservation group (L‐SPDP)	Robotic spleen‐preservation group (R‐SPDP)	Overall	*p* value
	(*n* = 57)	(*n* = 73)	(*n* = 130)	
Type of spleen preservation, *n* (%)				
‐Kimura	25 (64.1)	28 (59.6)	53 (61.6)	
‐Warshaw	14 (35.9)	4 (8.5)	18 (20.9)	0.445
‐Unknown	0	15 (31.9)	15 (17.5)	
Site of pancreas transection, *n* (%)			()	
‐Neck/isthmus	18 (31.6)	36 (49.3)	54 (41.5)	**0.050**
‐Body (further to the left)	39 (68.4)	37 (50.7)	76 (58.5)	
Stump closure method, *n* (%)				
‐Stapler	36 (63.2)	43 (58.9)	79 (60.7)	0.099
‐Suture	17 (29.8)	11 (15.1)	28 (21.5)	
‐Ultrasonic device	2 (3.5)	14 (19.2)	16 (12.3)	
‐Other	2 (3.5)	5 (6.8)	7 (5.5)	
Conversion to open, *n* (%)	11 (19.3)	3 (4.1)	14 (10.8)	**0.009**
Blood loss (mL), mean (SD)	317 (441)	151 (150)	221 (317)	**0.004**
Operative time (min), mean (SD)	312 (145)	284 (99)	296 (122)	0.200
Patients with any postoperative complication, *n* (%)	35 (61.4)	32 (43.8)	67 (51.5)	0.053
CCI, mean (SD)	15 (14.7)	8 (11.4)	11 (13.4)	**0.002**
CD ≥ III, *n* (%)	9 (15.8)	7 (9.6)	16 (12.3)	0.298
POPF grade B/C, *n* (%)	24 (42.1)	11 (15.1)	35 (26.9)	**0.001**
Time before drain removal, days, mean (SD)	35 (31)	11 (11)	21 (24)	**<** **0.001**
Postoperative haemorrhage, *n* (%)	2 (3.5)	3 (4.1)	5 (3.8)	1
Reoperation, *n* (%)	3 (5.3)	3 (4.1)	6 (4.6)	1
Time to regular diet, days, mean (SD)	6 (4.7)	3.7 (2.5)	4.7 (3.8)	**0.001**
Hospital stay, days, mean (SD)	7.5 (4)	7.1 (3.8)	7.3 (3.8)	0.628
Readmission, *n* (%)	6 (10.5)	8 (11)	14 (10.8)	0.585
Final pathology				
‐PNET	22 (38.6)	30 (41.1)	52 (40)	0.545
‐Mucinous cystic neoplasm	8 (14)	9 (12.4)	17 (13.1)	
‐Serous cystadenoma	4 (7)	13 (17.8)	17 (13.1)	
‐IPMN	3 (5.3)	5 (6.8)	8 (6.2)	
‐Solid pseudopapillary tumor	5 (8.8)	6 (8.2)	11 (8.5)	
‐Pseudocyst	4 (7)	2 (2.7)	6 (4.6)	
‐Other	11 (19.3)	8 (11)	19 (14.5)	

*Note:* In bold: statistically significant difference.

Abbreviations: CCI, Comprehensive Complication Index; CD, Clavien–Dindo; IPMN, intraductal papillary mucinous neoplasm; PNET, pancreatic neuroendocrine tumor; POPF, post‐operative pancreatic fistula; SD, standard deviation.

### 3.2 Postoperative Outcomes

Conversion to open surgery and blood loss were statistically significantly higher in the L‐SPDP versus R‐SPDP groups (19.3% vs. 4.1%, *p* = 0.009 and 317 vs. 151 mL, *p* = 0.004, respectively) (Table 2). Conversions in robotic surgery were as follows: adhesions [1], bleeding [1], and difficulty in achieving spleen preservation [1]. Operative times were similar between the groups.

Approximately 50% of the patients experienced at least one complication, without differences in terms of severe complications (CD grade III or higher) in each group. On the other hand, mean CCI index was higher in L‐SPDP than in the R‐SPDP group (15 vs. 8, *p* = 0.002). There was no mortality. Clinically relevant POPF was more frequent in the L‐SPDP group compared to the R‐SPDP group, 42.1% versus 15.1% respectively, reaching statistical significance (*p* = 0.001) (Table 2). The mean time to drain removal was threefold and time to regular diet was almost double in the L‐SPDP group compared with the R‐SPDP group (35 vs. 11 days, *p* < 0.001 and 6 vs. 3.7 days, *p* = 0.001, respectively). There were no differences in the term of reoperation, hospital length of stay, readmission rate, or final pathologic diagnosis (Table 2).

### 3.3 Univariate and Multivariate Analysis

Table 3 shows the univariate analysis of factors associated with clinically relevant POPF in the MI‐SPDP population. Preoperative baseline factors such as age and preoperative diabetes did not correlate with the probability of developing POPF. Technical elements related to the type of MI‐SPDP, conversion to open surgery, operative time, or final pathologic diagnosis were not associated with the development of grade B/C POFP at univariate analysis. ASA score III or higher was associated with POPF in univariate analysis. Other factors associated with POPF at univariate analysis were laparoscopic approach, pancreatic body transection and pancreatic stump closure method other than stapler. The multivariate model identified laparoscopic approach (OR 4: 95 CI 1.613–10.124; *p* = 0.003), pancreatic body transection (OR 2.6: 95 CI 1.00–6.973; *p* = 0.051) and pancreatic stump closure method other than stapler (OR 3.2: 95 CI 1.197–7.297; *p* = 0.05) as factors independently associated with clinically relevant POPF in obese population underwent MI‐SPDP (Table 3).

**TABLE 3:** Univariate and multivariate analysis of factors related to clinically relevant POPF.

	POPF grade B/C							
Variables	Univariate analysis				Multivariate analysis			
	*p*	OR	95% CI lower	95% CI upper	*p*	OR	95% CI lower	95% CI upper
Laparoscopic approach	**0.001**	4.2	1.810	9.558	**0.003**	4	1.613	10.124
Diabetes	0.413	1.6	0.505	5.270				
Pancreatic body transection	**0.004**	3.9	1.556	9.849	**0.051**	2.6	1	6.973
Male sex	0.077	2.1	0.925	4.564				
Stump closure method other than stapler	**0.004**	3.3	1.478	7.412	**0.019**	3.2	1.197	7.297
ASA ≥ III	**0.030**	2.6	1.097	6.133	0.385	1.5	0.587	3.979
Kimura spleen‐preservation	0.750	1.1	0.600	2.031				
Conversion to open	0.106	5.5	0.695	43.923				
Operative time	0.139	1	0.999	1.006				
Final histology PNET	0.702	0.855	0.384	1.905				

*Note:* In bold: statistically significant difference.

Abbreviations: AUC, area under the curve; CI, confidence interval; OR, odds ratio; PNET, pancreatic neuroendocrine tumor; POPF, post‐operative pancreatic fistula.

## 4 Discussion

In this study, we demonstrated that the robotic approach significantly reduces the incidence of postoperative pancreatic fistula (POPF) in obese patients undergoing spleen‐preserving distal pancreatectomy (MI‐SPDP) compared with the laparoscopic approach. In addition to the robotic approach, the use of stapler devices and the transection of the pancreas at the pancreatic neck were associated with lower rates of POPF.

Spleen‐preserving pancreatectomy is a surgical procedure intended to remove benign or low‐grade malignant tumours from the body and tail of the pancreas while sparing the spleen. This technique can help prevent complications that can arise from splenectomy, such as an increased susceptibility to infections and the risk of thromboembolic events [18]. The procedure is typically performed using either the vessel‐preserving technique (Kimura's) or Warshaw's technique [19]. Several studies have attempted to compare these two techniques, and while the first is associated with lower rates of spleen‐related postoperative complications, the latter seems easier to perform and could be associated with a higher rate of spleen preservation [19]. For this reason, some centres have described a stepwise approach to spleen preservation, adopting Warshaw's technique when Kimura's technique seems challenged [20].

In recent years, minimally invasive approaches, such as laparoscopic and robotic surgeries, have become increasingly favoured over conventional open surgery and these methods offer significant benefits, including less intraoperative bleeding and faster recovery [21]. Previous studies indicate that robotic surgery provides superior precision and lower rates of conversion to open surgery compared with laparoscopy [6]. Nonetheless, results concerning spleen preservation and complication rates can differ, and the choice between techniques often depends on the surgeon's skill and the individual patient's needs. Ultimately, while the goal of spleen‐preserving pancreatectomy is to mitigate the risks associated with splenectomy, success relies heavily on precise patient selection and meticulous surgical planning. In this context, obese patients represent a challenge for surgeons. We have previously demonstrated that the robotic approach favours the possibility of completing the operation by minimally invasive surgery, resulting in potentially lower complications in these patients [11, 14]. However, in our study, we also demonstrated that the robotic approach has a beneficial impact on postoperative pancreatic fistula (POPF). It is difficult to explain the reason for this result; however, the robotic approach in obese patients clearly allows for less manipulation of the pancreas, improved exposure of the surgical field, and better control of bleeding events, thereby avoiding manoeuvres that can traumatize the pancreatic parenchyma.

It is important to emphasise that spleen preservation in obese patients is a challenging operation. Previous studies have shown that spleen preservation in non‐obese patients is higher than 80% [22]. In our study, the spleen could only be preserved in 66.2% of the initially planned population and there was no significant difference between laparoscopic and robotic approaches (68.4% vs. 64.4%). However, this result should also be read in the context of a much higher conversion rate in the laparoscopic group, meaning that some patients may have been converted to open surgery in order to achieve spleen preservation. Unfortunately, we had insufficient data to investigate this finding in detail.

Transection of the pancreas at the neck was an important factor associated with lower rates of POPF. This could be due to the decreased thickness of the pancreas at this site and the lower quantity of pancreatic parenchyma left behind following the operation. Although this is not a new finding, it is important to emphasize the message that pancreatic resection should not be systematically extended to the neck. However, it should certainly be taken into account that there might be a higher possibility of CR‐POPF when the pancreas is transected more distally. This is particularly important when operating on patients at high risk for complications and also when deciding which strategies to put in place to mitigate the potential effect of a POPF, such as postoperative ICU stay, intrabdominal drain, enhanced recovery pattern, and systematic use of postoperative imaging, etc. Furthermore, in our cohort, the laparoscopic approach showed a higher rate of transection of the pancreas further to the left. These data could influence the development of POPF in the LDP group and increase its incidence, not only due to the surgical approach. However, according to the multivariate analysis, this is unlikely as both factors have an independent effect. To avoid this bias, we performed a stratified analysis according to the site of pancreatic transection, which showed that the laparoscopic approach was still the most important factor influencing the development of POPF.

In the case of left pancreatic resection, De Pastena et al. described that pancreatic neck thickness and pancreatic duct diameter are important preoperative variables that should be considered when assessing the individual risk of POPF [23]. Adding intraoperative variables such as body mass index, pancreatic texture, and operative time allows categorization into three risk groups of POPF (low, intermediate, and high). Elevated pancreatic fistula scores (PFS) are associated with prolonged hospital stays, increased risk of secondary infections, and a higher likelihood of additional interventions. Unfortunately, we could not calculate the PFS. This is a crucial tool for assessing the severity of pancreatic fistulas following distal pancreatectomy and helps predict the clinical significance of postoperative pancreatic leaks by evaluating several key factors. However, data on pancreatic texture were not available, and it would be very difficult to extract this information, especially in robotic surgery when tactile feedback is not available. Although a proper risk score could not be estimated due to the retrospective nature of the study, the population studied clearly represents a high‐risk group of patients due to the high BMI, high operative time, and mostly benign indications which are often associated with non‐dilated pancreatic duct and soft pancreatic texture. Other authors have demonstrated that several intraoperative variables should also be considered, such as stapler devices, type of cartridge, suture of pancreatic stump, and anastomosis. In our study, the stapler device was clearly associated with a lower risk of POPF, when compared to other closure methods including suture reinforcement. This association has been previously described by other authors and is consistent with experiences from high‐volume centres. However, these studies have mainly focused on left pancreatectomy with splenectomy, and there are no studies assessing the risk factors of POPF following spleen preservation. Unfortunately, the role of laparoscopic versus robotic stapler in terms of handling possibly associated with POPF could not be assesed as in many centres laparoscopic staplers are used during robotic surgery to reduce costs.

This study has several limitations: firstly, it is a retrospective study, and the original database was not collected for the specific purpose of this analysis. Also, the sample size was relatively small; however, this is the largest study ever performed on spleen preservation in obese patients using a minimally invasive approach, as this type of surgery is not commonly performed in this setting. Unfortunately, there was a lot of missing information on the type of spleen preservation performed, which made it impossible to assess the effect of a specific technique on the main outcome of the study. Finally, another important limitation is that the role of avoiding the systematic use of abdominal drains, as recently suggested by the PANDORINA trial, remains unexplored [24]. The issue of these patients requiring bariatric surgery in the future was not addressed in the current study. The Warshaw technique, which relies on short gastric vessels for splenic perfusion, may have implications for future surgical planning, particularly for sleeve surgery. This should be carefully considered when performing spleen preservation.

In conclusion, the robotic approach significantly reduces the POPF occurrence in obese patients undergoing MI‐SPDP compared with the laparoscopic approach. Further larger series are needed to confirm our findings.

## Author Contributions

**Fabio Ausania:** conceptualization, data curation, formal analysis, supervision, writing, and hyphenation, review and editing. **Filippo Landi:** conceptualization, data curation, formal analysis. **Carolina González‐Abós:** conceptualization, data curation, formal analysis, writing. **John B. Martinie:** methodology, resources. **Dionisios Vrochides:** methodology, resources. **Matthew Walsh:** methodology, resources. **Shanaz M. Hossain:** methodology, resources. **Steven White:** methodology, resources. **Viswakumar Prabakaran:** methodology, resources. **Laleh G. Melstrom:** methodology, resources. **Yuman Fong:** methodology, resources. **Giovanni Butturini:** methodology, resources. **Laura Bignotto:** methodology, resources. **Valentina Valle:** methodology, resources. **Yuntao Bing:** methodology, resources. **Dianrong Xiu:** methodology, resources. **Gregorio Di Franco:** methodology, resources. **Francisco Sanchez‐Bueno:** methodology, resources. **Nicola de'Angelis:** methodology, resources. **Alexis Laurent:** methodology, resources. **Giuseppe Giuliani:** methodology, resources. **Graziano Pernazza:** methodology, resources. **Alessandro Esposito:** methodology, resources. **Roberto Salvia:** methodology, resources. **Francesca Bazzocchi:** methodology, resources. **Ludovica Esposito:** methodology, resources. **Andrea Pietrabissa:** methodology, resources. **Luigi Pugliese:** methodology, resources. **José Rios:** methodology. **Andrea Coratti:** methodology, resources. **Luca Morelli:** validation. **Pier C. Giulianotti:** validation.

## Ethics Statement

Approval of the institutional review board of our Institution (CEIm, Comité de Ética de la Investigación con medicamentos) with identification number HCB/2022/0438 was obtained.

## Conflicts of Interest

The authors declare no conflicts of interest.

## Data Availability

The data that support the findings of this study are available on request from the corresponding author. The data are not publicly available due to privacy or ethical restrictions.
